# Chicken Embryonic-Stem Cells Are Permissive to Poxvirus Recombinant Vaccine Vectors

**DOI:** 10.3390/genes10030237

**Published:** 2019-03-20

**Authors:** Efstathios S. Giotis, Guillaume Montillet, Bertrand Pain, Michael A. Skinner

**Affiliations:** 1 Section of Virology, Department of Medicine, St Mary’s Campus, Imperial College London, Norfolk Place, London W2 1PG, UK; 2 Univ Lyon, Université Lyon 1, INSERM, INRA, Stem Cell and Brain Research Institute, U1208, USC1361 Bron, France; guillaume.montillet@inserm.fr (G.M.); bertrand.pain@inserm.fr (B.P.)

**Keywords:** embryonic stem cells, RNA-sequencing, pluripotency, recombinant vaccine viruses

## Abstract

The discovery of mammalian pluripotent embryonic stem cells (ESC) has revolutionised cell research and regenerative medicine. More recently discovered chicken ESC (cESC), though less intensively studied, are increasingly popular as vaccine substrates due to a dearth of avian cell lines. Information on the comparative performance of cESC with common vaccine viruses is limited. Using RNA-sequencing, we compared cESC transcriptional programmes elicited by stimulation with chicken type I interferon or infection with vaccine viruses routinely propagated in primary chicken embryo fibroblasts (CEF). We used poxviruses (fowlpox virus (FWPV) FP9, canarypox virus (CNPV), and modified vaccinia virus Ankara (MVA)) and a birnavirus (infectious bursal disease virus (IBDV) PBG98). Interferon-stimulated genes (ISGs) were induced in cESC to levels comparable to those in CEF and immortalised chicken fibroblast DF-1 cells. cESC are permissive (with distinct host transcriptional responses) to MVA, FP9, and CNPV but, surprisingly, not to PBG98. MVA, CNPV, and FP9 suppressed innate immune responses, while PBG98 induced a subset of ISGs. Dysregulation of signalling pathways (i.e., NFκB, TRAF) was observed, which might affect immune responses and viral replication. In conclusion, we show that cESC are an attractive alternative substrate to study and propagate poxvirus recombinant vaccine vectors.

## 1. Introduction

Despite advances in cell line development, embryonated eggs and primary chicken embryonic fibroblasts (CEF) derived from them remain the main avian substrate for the study of avian viruses and the production of vaccines (both avian and human). However, their use is associated with limitations [1] such as high cost, security of supply (being vulnerable, for instance, to outbreaks of avian influenza in poultry), batch-to-batch reproducibility, risk of adventitious infections, and lengthy production processes. Pluripotential stem cell lines, which are relatively stable genetically and have an unlimited life span, can potentially offer a quicker, more secure, more cost-effective, and higher-yield cell culture system [2,3,4,5]. 

First identified in mice and later in other mammals, embryonic stem cells (ESC) have also been isolated in birds. Chicken ESC (cESC) have been derived from cultures of blastodermal cells taken from stage X-XIII chick embryos [6,7]. They are positive for alkaline phosphatase and the SSEA1 antigen, and can differentiate into various cell types in vitro or when injected into recipient embryos [7,8]. cESC can be grown and maintained relatively easy in culture and can sustain their phenotypic characteristics after extensive expansion [7,9]. 

The unique self-renewal features and lack of transforming oncogenes and adventitious agents render ESC ideal cell substrates for the manufacture of viral vaccines. The vaccine industry has a long and extensive history of using avian substrates so, with the ethical and safety issues associated with mammalian ESC, there has been a shift of focus towards employing avian ESC for vaccine production. For instance, the duck embryonic stem-cell line EB66 (Valneva, Saint-Herblain, France) has received market authorisation from the Japanese health authorities for H5N1 influenza vaccine production [10]. Furthermore, the cESC EBx cell lines EB14 and EB45 (Vivalis, Saint-Herblain, France) showed promising cell growth in bioreactors and productivities for a wide range of viruses [11]. 

The limited availability of continuous, spontaneously immortalised cell lines for diagnosis and research has been a critical problem in the field of avian virology. Currently, the standard avian cell substrates for studies on avian viruses are primary CEF and the CEF-derived, spontaneously immortalised DF-1 fibroblast cell line. There is a growing need for more in vitro cell culture systems that support the infection of avian viruses and accurately reiterate virus-host interactions. cESC can provide a physiologically relevant in vitro model system for studying host responses to avian and mammalian wild or vaccine viruses [7,8]. They have been compared with chicken blastodermal and germ cells at gene expression level, using microarray analysis, and were found to express classic pluripotency-related genes (including *OCT4*//*POU5F3*, *NANOG*, and *SOX2*/*3*) [12,13]. They are also known for their ability to replicate a small number of viruses of interest to the vaccine industry [7,8,14]. Their response to more representative wild or vaccine avian viruses remains uncharacterised. It is also unclear whether these cells are able to launch an interferon response upon infection with immunogenic viruses or in response to exogenous type I interferon (IFN; which we refer to as chIFN-α, for its familiarity in mammalian systems, even though it is not standard nomenclature in avian species). 

We asked whether pluripotent cESC are susceptible to infection by a panel of four vaccine viruses, specifically three poxviruses—modified vaccinia virus Ankara (MVA), fowlpox virus (FWPV), canarypox virus (CNPV)—and one birnavirus, infectious bursal disease virus (IBDV). Viruses typically employ the host cellular machinery to translate viral mRNA, and hijack host immune defenses in order to replicate. We aimed to identify with RNA-sequencing the genes involved in the cESC-specific innate immune response, and determine restriction or permissiveness toward virus infection and replication. Characterising changes in the global transcriptional profiles in response to viral infection can provide insights into the regulatory interplay between cESC and viruses. The emerging picture is that of a diversity of patterns of gene expression upon viral infection. We show that cESC are responsive to exogenous recombinant chIFN-α in terms of general interferon regulated genes (IRGs) mRNA expression and are permissive to MVA, FP9, and CNPV but not to IBDV PBG98. Interestingly, cESC showed higher production of MVA than DF-1 cells. Collectively, these data suggest that cESC can serve as a high-yielding cell line for the study and propagation of vaccine viruses.

## 2. Materials and Methods

### 2.1. Cell Culture

cESC were established from blastodermal cells and amplified on mouse STO feeder cells, irradiated using a Nordion gammacell irradiator (as described previously [6,8]). cESC were propagated in DMEM/F12 (Gibco/Fisher Scientific, Loughborough, UK) medium containing 10% non-heat inactivated foetal bovine serum (Techgen batch no F 93246, Les Ulis, France), 2% chicken serum (Valbiotech, Paris, France), 1% sodium pyruvate (100 mM; Gibco/Fisher Scientific), 1% glutamine (200 mM; Gibco/Fisher Scientific), 1% non-essential amino acid (Gibco/Fisher Scientific), 0.16 mM β-mercaptoethanol (Sigma, St. Louis, MO, USA), 1% penicillin/streptomycin (Gibco/Fisher Scientific) supplemented with human-insulin-like groth factor (IGF-1; 5 ng/mL), human-stem cell factor (SCF; (1 ng/mL), human interleukin 6 (IL6; 1 ng/mL), human interleukin 6 receptor alpha (IL6 Ra; 1 ng/mL), and mouse-leukemia inhibitory factor (LIF; 1000 U/mL; all from Peprotech, London, UK). Freshly isolated CEFs were provided by The Pirbright Institute (Surrey, UK). Cells were seeded in T25 flasks (Greiner Bio One; 5.6 × 10^6^ cells/flask) and cultured overnight in 5.5 mL 199 media (Gibco, Invitrogen, Carlsbad, CA, USA) supplemented with 8% heat-inactivated newborn bovine serum (NBCS; Gibco, Invitrogen), 10% tryptose phosphate broth (TPB; Sigma, Gillingham, UK), 2% nystatin (Sigma), and 0.1% penicillin and streptomycin (Gibco, Invitrogen, Paisley, UK). DF-1 were propagated in Dulbecco’s minimal essential medium (DMEM) (Life Technologies, Carlsbad, CA, USA) supplemented with 10% heat-inactivated fetal bovine serum (Life Technologies) and penicillin and streptomycin. All cell cultures in this study were maintained at 37 °C and 5% CO_2_.

### 2.2. Confirmation of Pluripotency

For confirmation of expression of the glycolipid antigen SSEA1 (which is typically overexpressed in pluripotential chicken cells), cells were washed twice in ice-cold FACS buffer and stained with an unconjugated anti-SSEA1 mouse monoclonal antibody IgM for 30 min at 4 °C (MC-480, Chemicon, Moses Lake, WA, USA) and then with a fluorescein isothiocyanate (FITC)-labelled goat anti-mouse IgM secondary antibody (F9259, Sigma). The BD LSR Fortressa was used to determine expression of SSEA1, in comparison to the matched unstained control. SYTOX stain (ThermoFisher, Loughborough, UK) was used for cell viability discrimination and the data files were analysed using FlowJo software v10.0 (Tree Star, San Carlos, CA, USA., BD Biosciences, Wokingham, UK). Experiments were carried out twice. Cell morphology imaging was conducted using the Evos FL microscope (Thermofisher).

### 2.3. Virus Propagation, Titration, and Purification

Viruses used in this study were: (i) FP9, a high passage-attenuated European strain of FWPV (the prototypic member of the Avipoxviruses) [15], which has been used as a recombinant vector for the expression of antigens from several avian and human pathogens [16]; (ii) a commercial CNPV vaccine strain (Duphar, Fort Dodge) [17]; (iii) MVA, a human vaccine virus which was developed by long-term serial passage on CEF (such that it is now unable to propagate on the vast majority of mammalian cell lines) for use as a safe vaccine against human smallpox [18]; or (iv) PBG98, a commercial, CEF-adapted, attenuated vaccine strain of the avibirnavirus, IBDV [19]. Confluent CEF were infected with each virus at 0.01 PFU per cell. At 5 days post infection (p.i.), the supernatant and remaining cells were harvested. This suspension was freeze-thawed thrice and clarified for 30 min at 1000× *g*. For poxviruses, the supernatant was centrifuged for 1 h at 40,000× *g* and the pellet was resuspended in TMN buffer (10 mM Tris (pH 7.5), 1.5 mM MgCl_2_, 10 mM NaCl) and centrifuged for 2 h at 160,000× *g* through a 25% (*w*/*w*) sucrose cushion in TMN buffer. The pellet was resuspended in 1 mL of TMN buffer and sonicated before being layered over a sucrose gradient (15–40% *w*/*w*) and centrifuged at 30,000× *g* for 50 min. The resulting virus band(s) was removed by side puncture of the tube using a needle and syringe, and the virus concentrated by centrifugation at 40,000× *g*. To visualise plaques clearly, an additional neutral red overlay media (MEM + 2% NBBS + 1% low melting point agarose + 1% neutral red) was applied and incubation was carried out for a further 24 h. IBDV PBG98 was purified through a cushion of 30% (*w*/*w*) sucrose as previously described [20].

### 2.4. Viral Infections and chIFN-α Stimulation

Fully confluent cESC, and DF-1 grown in T25 flasks were washed with phosphate buffer saline (PBS) and infected for 2 h with each one of the viruses (at multiplicity of infection, MOI, of 0.01 or 5) or mock-infected. The inoculum was then removed and cells were washed and further incubated in maintenance medium for 14 h (MOI: 5) until cell collection/RNA isolation (for qRT-PCR/RNA-seq) or for 96 h (MOI: 0.01) post infection (p.i.) for determination of viral titre. Viral titres in cESC and DF-1 were determined by classical plaque assay of their supernatants in CEFs (96 h p.i.) as previously [21]. For chIFN-α stimulation experiments, recombinant chicken chIFN-α was prepared as previously reported [22] and was added in culture media to a final concentration of 1000 U/mL. Cells were dissociated with pronase (from *Streptomyces griseus*, Sigma) and stored at −80 °C in RNALater (Sigma) until RNA extraction. Experiments were conducted in triplicate.

### 2.5. RNA Extraction and Processing of Samples for RNA-Sequencing

Total RNA was extracted and processed from mock- and virus infected-, and mock- and chIFN-α- stimulated cESC using an RNeasy kit (Qiagen, Hilden, Germany) as previously [23]. On-column DNA digestion was performed using RNase-free DNase (Qiagen) to remove contaminating genomic DNA. RNA samples were quantified using a Nanodrop Spectrophotometer (Thermo Scientific, Waltham, MA, USA) and checked for quality using a 2100 Bioanalyzer (Agilent Technologies, Santa Clara, CA, USA). All RNA samples had an RNA integrity number (RIN) ≥ 9.6.

### 2.6. IIIumina cDNA Library Preparation and Sequencing

Triplicate samples from each treatment were pooled for library production and strand-specific RNA-sequencing at the Beijing Genomics Institute (BGI, Hong Kong). Poly (A) mRNA was purified from total RNA using magnetic beads with oligo (dT) (New England BioLabs, Hitchin, UK). The mRNA was then broken into short fragments using divalent cations at an elevated temperature. Using these cleaved RNA fragments as templates, random hexamer primers (Invitrogen) was used for first-strand cDNA synthesis, followed by second-strand cDNA synthesis using DNA polymerase I (New England BioLabs) and RNase H (Invitrogen). Libraries were sequenced using the Illumina HiSeq2000 system.

RNA-seq data were imported into CLC bio’s Genomics Workbench (CLC bio, Qiagen Bioinformatics, Aarhus, Denmark), quality-controlled, and thereafter processed using that package (versions 7.5.1). After quality control, the reads were subjected to quality trimming then mapped against ENSEMBL galGal4 annotated genes (release 82) for quantitative analysis of expression. Fold change and False Discovery Rates (FDR) were calculated using Kal’s Z test [24], with pooled data. RNA-seq data are deposited in the GEO and SRA archives at NCBI, with the accession number (GSE127203). Full data summaries are listed in Supplementary file 1. Comparisons were also made with a previously published interferon-stimulated genes (ISG) dataset [25] (ENA study: PRJEB7620), analysed in an identical manner.

### 2.7. Bioinformatic Analyses

Data mining and enrichment analysis was performed using Genego, part of the MetaCore software suite (Clarivate Analytics, Philadelphia, PA, USA). Enrichment analysis consisted of mapping chicken gene IDs from the datasets onto gene IDs of human orthologues as entities of built-in functional ontologies represented in MetaCore by pathway maps and process networks. Statistical significance was measured by the number of genes that map onto a given pathway and was calculated on the basis of *p*-value, based on hypergeometric distribution (a built-in feature of MetaCore). Full enrichment analysis (enrichment by Gene Ontology (GO) processes, process networks, pathway maps, and protein function) identified using the Metacore transcription regulation algorithm is presented in Supplementary file 2. Venn diagrams were generated using the ADHoRe suite [26].

### 2.8. Quantitative Real-Time RT PCR

Quantitative real-time RT PCR was performed on RNA samples using a two-step procedure. RNA was first reverse-transcribed into cDNA using the QuantiTect Reverse Transcription Kit (Qiagen) according to manufacturer’s instructions. qPCR was then conducted on the cDNA in a 384-well plate with a ABI-7900HT Fast qPCR system (Applied Biosystems, Foster City, CA, USA). Mesa Green qPCR MasterMix (Eurogentec, Liège, Belgium) was added to the cDNA (5 μL for every 2 μL of cDNA). The following amplification conditions were used: 95 °C for 5 min; 40 cycles of 95 °C for 15 s, 57 °C for 20 s, and 72 °C for 20 s; 95 °C for 15 s; 60 °C for 15 s; and 95 °C for 15 s. Primer sequences for genes that were used in the study are given in Supplementary file 3. The output Ct values and dissociation curves were analysed using SDS v2.3 and RQ Manager v1.2 (Applied Biosystems). Gene expression data were normalised against the 40 s ribosomal protein S17 (*RS17*) housekeeping gene, and compared with mock controls using the comparative C_T_ method (also referred to as the 2^−ΔΔCT^ method [27]). All samples were loaded in triplicate.

### 2.9. Statistical Analyses

To determine the significance of differences between experimental groups, one-way ANOVA *t*-tests were performed using the fold change scores with a Bonferroni multiple comparisons test. *p*-values were set at 0.05 (*p* ≤ 0.05) unless indicated otherwise. Error bars represent the standard error of the mean (SEM) or standard deviation (SD). The correlation of expression values between RNA-seq analysis and qRT-PCR was statistically assessed by calculation of Pearson’s correlation coefficient using the built-in function of GraphPad Prism (v.6.0).

## 3. Results and Discussion

### 3.1. Confirmation of the Pluripotent Status of cESC

**Figure 1:**
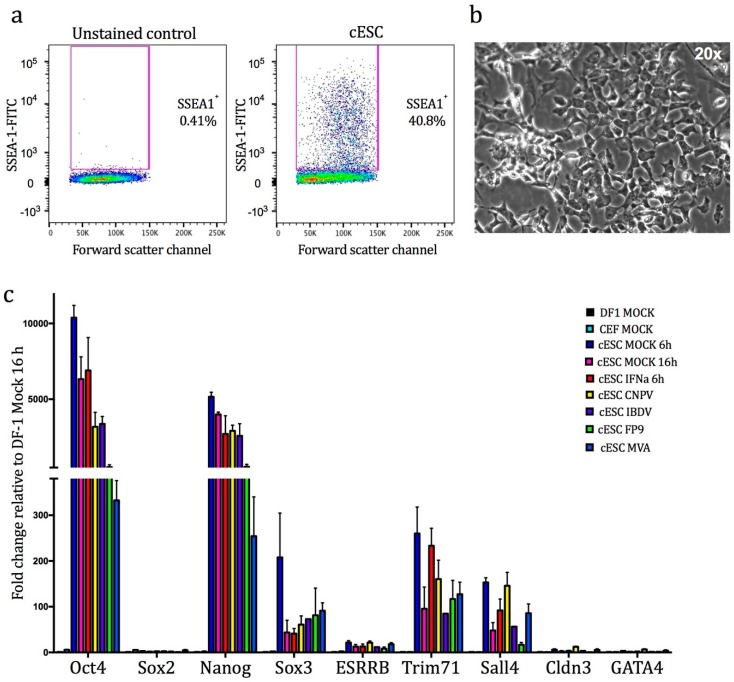
(**a**) Forward scatter plotted versus anti-stage specific embryonic antigen SSEA1–fluorescein isothiocyanate (FITC) fluorescence for cESC showing an identifiable SSEA1–positive cESC sub-population. Left: Unstained control. (**b**) Phase contrast microscopy image of chicken embryonic stem cells (cESC) culture (magnification 20×; Evos Fl microscope). (**c**) Fold change in expression by qRT-PCR of cESC pluripotency markers during viral infection and stimulation with recombinant chIFN-α. Values are normalised against DF-1 mock-infected cells. Error bars represent SEM (*n* = 3).

Embryonic stem cells are pluripotent, meaning they have the potential to differentiate into multiple cell types. Chicken embryonic stem cells (cESC) are propagated in a pluripotent ‘undifferentiated’ state on inactivated (by means of irradiation) feeder cells (mouse embryonic fibroblast cells (STO)) and exhibit morphology similar to mouse ESC [28]. The culture conditions that support the undifferentiated phenotype are well-documented and include the use of growth factors and cytokines such as mouse LIF, and a combination of human IL-6/SCF/IGF-1/IL-6Ra in the medium [9]. The undifferentiated status of cESC was confirmed by flow cytometry for the pluripotency marker, surface antigen SSEA1. This analysis confirmed the existence of an identifiable SSEA1–positive cESC cell sub-population unlike the unstained control (typical heterogeneity: ~40% of the population, Figure 1a). ESC have the propensity for spontaneous differentiation to other embryonic lineages upon formation of cell aggregates called embryoid bodies. Microscopic analysis of cESC cultures confirmed the lack of such formations. Furthermore, cells were able to generate characteristic cESC colonies of small cells, tightly packed in nests with typical “ES-like” morphological features (Figure 1b, [6]). These findings confirm that cells maintained their pluripotential phenotype and did not differentiate into somatic lineages.

We aimed to assess permissiveness and host gene expression of cESC in response to infection with a panel of vaccine viruses. To minimise the effect of feeder cells, which can affect gene expression studies [29], cESC were expanded without them for two passages. Cells were then infected (MOI: 5) with purified suspensions of FWPV FP9, CNPV, MVA, or IBDV PBG98. A pilot time-course study was initially conducted with qRT-PCR (0, 4, 8, 16, and 24 h p.i.; data not shown) to identify the optimal time for collection of infected cells. Total RNA was isolated from each of these samples and evaluated for quality and quantity using standard techniques. For subsequent studies, a 16 h p.i. time point was selected as: (i) Most changes in gene expression occurred at 16 h p.i, (ii) 16 h coincides with the approximate completion of the replication cycle of the viruses used in the study, and (iii) RNAseq/microarray data are available on infection of CEF and DF-1 at 16 h p.i. with the same viruses, allowing comparative analysis ([30] and unpublished data).

The IFN response is the first line of host defence against invading viruses and varies in different cell types and species [31]. To compare signature responses of cESC to chIFN-α and viral infection, we charted the cellular transcriptional response to exogenous recombinant chicken IFN-α (1000 U/mL) for 6 h. The resulting dataset was statistically integrated with a published dataset from chIFN-α-stimulated CEF [25]. Mock-stimulated and mock-infected samples were collected at both time points used in the study (6 and 16 h).

To assess whether treatment altered the differentiation status of cESC, we conducted a qRT-PCR for nine pleiotropically-acting transcription factors that are commonly used as markers for molecular characterisation of undifferentiated human, mouse, and chicken ESC (using non-cESC, DF-1 fibroblast cells as a reference; Figure 1c). *OCT4*/*POU5F3*, *SOX2*, and *NANOG* are considered the core transcriptional regulators of pluripotency [32]. Our results show that mock- and treated-cESC express high levels of *OCT4*/*POU5F3* and *NANOG* (but not *SOX2*, *ESRRB*, *CLDN3*, and *GATA4*) compared to untreated somatic chicken cells (DF-1 and CEF), in concordance with previous studies [12]. Conversely, cESC expressed higher levels of *SOX3*, *TRIM71*, and *SALL4* compared to somatic cells. Of note, viral infection of cESC (especially by FP9 and MVA) significantly reduced the expression levels of both *OCT4*/*POU5F3* and *NANOG* (Supplementary file 1).

### 3.2. Illumina 100b Paired-End Sequencing and Sequence Quality Control

**Figure 2:**
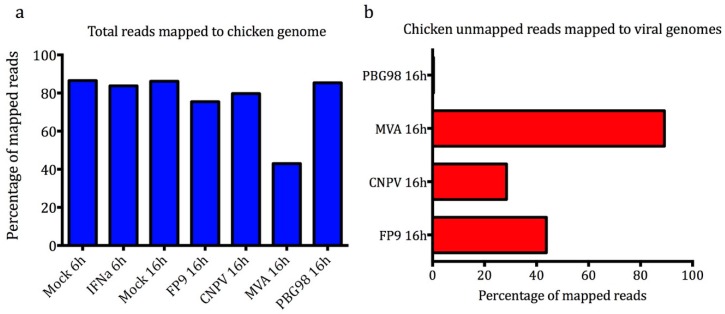
(**a**) Percentages of total reads mapped to chicken reference genome galGal4.82. (**b**) Percentages of the remaining (chicken-unmapped) reads mapping to viral reference genomes. Analysis was conducted using CLC bio’s Genomics Workbench (Qiagen) as indicated in Materials and Methods.

We undertook transcriptional profiling with RNA-seq (HiSeq Illumina 100 base paired-end sequencing) of cESC following chIFN-α-stimulation or viral infection. Data were analysed with CLC bio’s Genomics Workbench commercial software suite. The imported sequences, which had been trimmed for adapter sequences using Illumina software, were subjected to quality control using parameters such as per-sequence analysis, per-base analysis, and over-representation analysis. Reads were filtered and trimmed for ambiguity and low quality. Over 40 million clean reads were generated for each sample analysed and subjected to further downstream analysis. These high-quality processed paired-end reads were initially mapped to chicken reference genome (galGal4 release 82) and the residual unmapped reads (for the infected samples) were mapped to the corresponding virus reference genomes (CNPV AY318871; FWPV FP9 AJ581527; MVA AY603355; IBDV PBG98 AF194428; and AF194429). The results of the mapping showed that 86% of the reads from mock-treated samples mapped to the chicken genome (Figure 2a). The percentage of reads mapping to the chicken genome was lower for the infected samples, where PBG98 (roughly equivalent to Mock) > CNPV > FP9 >> MVA (with the last falling to about 40%). The percentage of those unmapped reads mapping to poxviral genomes ranged from 28% for CNPV to 89% for MVA but was only 0.3% for PBG98.

### 3.3. cESC Are Permissive to MVA, FP9, and CNPV But Not IBDV PBG98

Mapping of the residual reads (which failed to map to the chicken genome) to the respective reference virus genomes showed an inverse correlation between the percentage of virus-mapped reads (where MVA >> FP9 > CNPV >>> PBG98) and host-mapped reads (Figure 2b). The virus-mapped reads represent RNA expression for the poxviruses and RNA expression/replication for IBDV. They say nothing about genome replication for the poxviruses nor about production (and release) of infectious virus for any of the viruses. However, the results are consistent with the cES being potentially highly permissive to MVA, permissive to FP9 and CNPV but, given the near lack of RNA expression, restrictive to PBG98.

**Figure 3:**
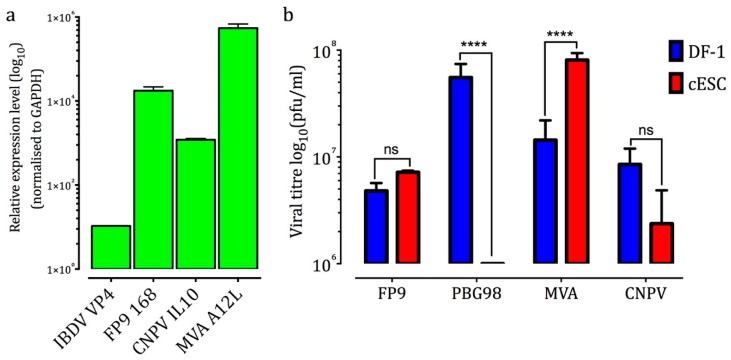
(**a**) Fold change of viral genes (related to mock-infected samples and normalised against housekeeping gene *RS17*) following viral infection of cESC with FP9, CNPV, MVA, and PBG98 (MOI: 5, 16 h p.i.). Data were calculated with qRT-PCR and are representative from three independent experiments. (**b**) Viral titres recovered from cESC and DF-1 after infection with the viruses (MOI: 0.1, 96 h p.i). Virus titres correspond to extracellular virus, determined by plaque assay on CEFs (Mean ± SD). Data are representative of two independent experiments. One-way ANOVA with a Bonferroni post hoc test was used to analyse the data. ns (not statistically significant): *p* > 0.05, ****: *p* < 0.0001.

Viral genome replication was assessed by qRT-PCR for expression of poxviral late (i.e., post-replicative) genes (CNPV *IL10*, FWPV *FPV168*, and vaccinia virus (VACV) *A12*). Only low levels of reads were detected for IBDV (*VP4* gene); they are negative-sense so are specific for the plus strand and could therefore represent transcripts or the plus strand of the double-stranded genome (Figure 3a). Productive replication of infectious MVA, FP9, and CNPV at 96 h p.i. (MOI: 0.1) was assessed by plaque assays of the supernatants of infected cESC. Viral yields were compared to those found in infected DF-1 (Figure 3b). MVA yield was 5.6 fold higher in cESC compared to DF-1 cells. Statistically, there was no significant difference in titres of FP9 and CNPV from cESC and DF-1 cells. No significant titre was detected for PBG98 in cESC, whereas DF-1 cells were highly permissive [30].

Having established that cESC can support productive infection for three of our tested viruses, we assayed the host transcriptome with RNA-sequencing. The fold change in gene expression was calculated based on the number of gene-mapped reads and normalised as RPKM (Reads Per Kilobase of transcript per Million mapped reads). Kal’s Z test was applied to test the significance of fold change (FC) for comparisons between virus-infected and mock samples. Comparisons were conducted between virus-infected and chIFN-α stimulated samples and their respective mock-infected or mock-stimulated samples (Supplementary file 1). A summary of the number of up- and down-regulated transcripts (FDR ≤ 0.05%, FC ≥ (±)1.5) is provided in Table 1. For the comparison between chIFN-α-stimulated and mock samples, a 6 h time point was chosen as it has been widely used and is known to result in significant levels of a broad range of IFN regulated genes (IRGs) in mammals and chicken [25]. Also, a more conservative fold change cut-off (FC ≥ (±)3.0) was used for consistency with a previous RNA-seq analysis, allowing direct comparisons with an established set of chicken IRGs [25].

### 3.4. cESC are Competent for High-Level IFN-Mediated Induction of ISGs

In birds, as in mammals, IFNs trigger tyrosine phosphorylation and activation of members of the Janus kinase (JAK) family of cytoplasmic tyrosine kinases that then activate the phosphorylation of signal transducers and activator of transcription, STAT1 and STAT2 [25]. Dimerisation of phosphorylated STATs triggers a cascade of events that ultimately leads to the transcription of hundreds of IFN-stimulated genes (ISGs), which mediate a range of key cellular processes including antiviral and other immune functions [33,34,35].

**Figure 4:**
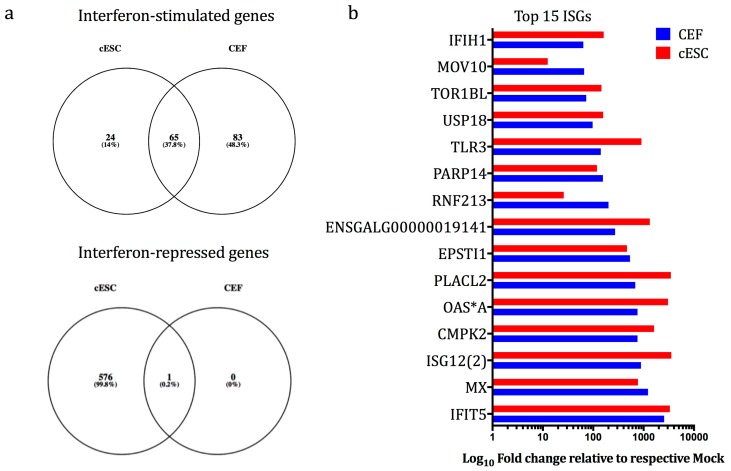
(**a**) Venn diagrams showing the distribution of unique and shared up- and down-regulated transcripts in interferon (chIFN-α)-stimulated CEF and cESC. The numbers in the intersections of each circle represents the number of transcripts common to each condition (FDR ≤ 0.05 and fold change ≥±3.0). (**b**) Differential expression (log_10_ fold-change related to respective mock samples) of the top 15 ISGs (fold change ≥|±3.0| and FDR ≤ 0.05) as identified in cESC and CEFs [25] following chIFN-α stimulation (Supplementary file 1).

Treatment of cESC with chIFN-α modulated the expression of 667 transcripts (i.e., interferon regulated genes, IRGs; Table 1), of which 90 were up-regulated (i.e., interferon stimulated genes (ISGs) but many more (577) were down-regulated, showing a more complicated IFN-regulated transcriptomic profile than we have previously observed by RNA-seq and microarrays with CEF [25,30] and by microarrays with DF-1 cells [30]. Comparison of the lists of differentially expressed genes from chIFN-stimulated CEF and cESC, using the gene symbol as a compatible identifier, demonstrated that 65 ISGs were induced in both chIFN-α-stimulated CEF and cESC (Figure 4a). Conversely, 24 ISGs were induced only in cESC, and 83 only in CEF. Comparing the overlapping data, the top 15 ISGs (based on CEF) show generally high degrees of concordance in terms of identity and relative levels of induction by chIFN-α with disparity mainly for a small number of canonical ISGs (e.g., *SAMD9L*, *RSAD2*, *GVINP1*), due to the very low basal expression of these transcripts in untreated cESC (which returns NaN values).

### 3.5. The Vaccine Vector Viruses Induce Distinct Gene Expression Profiles in cESC

**Figure 5:**
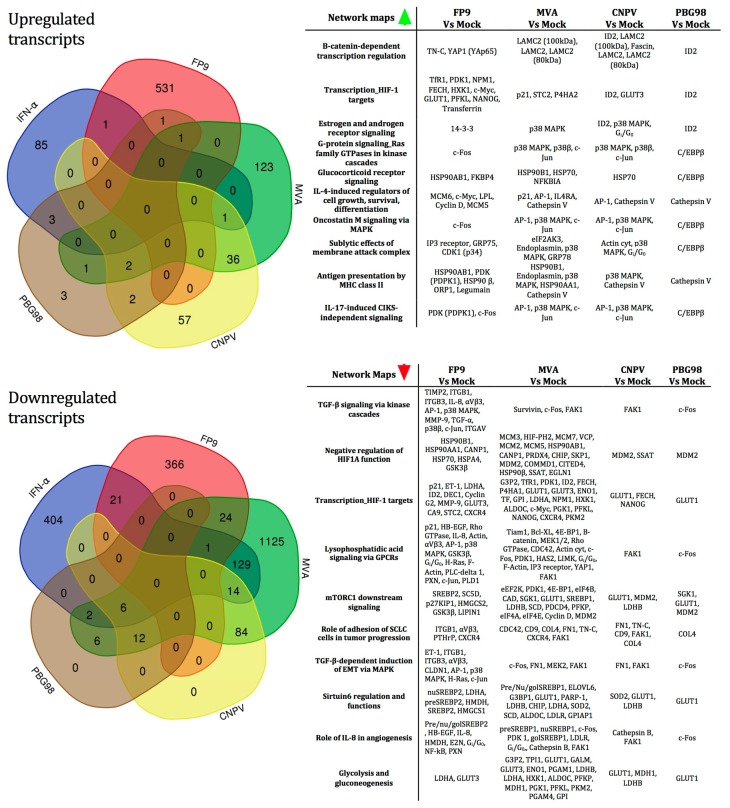
**Left:** Venn diagrams showing the distribution of unique and shared up- (top left panel) and down-regulated (lower left panel) transcripts in virus-infected cESC. The numbers in the intersections of each circle represent the number of transcripts common to each condition (FDR ≤ 0.05 and fold change ≥±1.5). **Right:** Top 10 statistically significant MetaCore GeneGO network pathway maps associated with up-regulated (top right table) and down-regulated transcripts in the comparison between virus-infected and mock-infected samples (Full enrichment analysis is described in Supplementary file 2).

We then compared the differential host gene expression induced by the four vaccine viruses. Full gene lists are provided in Supplementary file 1. Considering the absolute number of differentially regulated genes, MVA induces the most extensively modulated transcriptional programme among the viruses tested, with 164 transcripts up-regulated and 1403 transcripts down-regulated (Table 1). The high number of genes down-regulated by MVA is not entirely surprising and is consistent with previous studies, which have shown that orthopoxviruses (the genus of mammalian poxviruses that includes vaccinia, cowpox, and variola viruses) cause global switch-off of host gene transcription and translation [36]. Similar trends were observed for CNPV and PBG98. Conversely, FP9 up-regulates more transcripts than it down-regulates. Venn diagrams highlight the number of up- or down-regulated transcripts by the viruses (Figure 5), respectively, indicating that the majority of up-regulated transcripts are largely virus-specific. Compared to the up-regulated transcripts, the down-regulated subsets appear to be more highly shared, especially for CNPV and PBG98, indicating a possible general shut-down effect of the viruses on cESC replication and function (and possibly differentiation), of a type that we have not observed in CEF or DF-1.

Data were then processed using the GeneGo MetaCore pathway enrichment software and analysed for known ‘canonical’ pathways and biological functions (full enrichment analysis is presented in Supplementary file 2). The top 10 ranking canonical pathway maps associated with the up- and down-regulated transcripts in cESC following infection by all viruses are ranked in Figure 5 according to their *p*-value. Both up- and down-regulated transcripts were mainly associated with critical processes and signalling pathways involved in the maintenance of pluripotency as well as in the process of somatic cell reprogramming in mammalian cESC, such as the Wnt/β-catenin (*LAMC2*, *TN-C*), *TGF-β*, glycocorticoid receptor (*HSP70, HSP90*), and *MAPK* (p38) signalling pathways. Several transcripts, including those encoding the pleiotropic transcription factors *AP-1* (*c-FOS* and *c-JUN*) and *CdK1* as well as the negative regulator of cell differentiation, *ID2*, are involved in multiple cellular processes, such as differentiation, proliferation, and apoptosis.

Overall, the most abundant differentially regulated gene groups (>50 across all conditions tested in the study) were involved in *HIF1α*-mediated hypoxic signalling. Hypoxic microenvironment is an important stem cell niche [37,38,39]. In mouse ESC, both *HIF1α* and *HIF2α* are expressed; *HIF1α* appears to be central to regulating hypoxic responses [37], but it is *HIF2α* that regulates *OCT4*//*POU5F3* and stem cell maintenance [38]. In human ESC, the mechanism of hypoxic regulation appears to differ since HIF1α protein is only transiently expressed following exposure to low oxygen stress [39]. The mechanism(s) of action of *HIF1α* and *HIF2α* in cESC is not clear from this study’s data.

Despite the activation of the MAPK pathway and the high expression levels of transcription factor *AP-1* (both of which are positively involved in the expression of the pro-inflammatory cytokines), there is a general lack of cytokine and inflammation responses upon viral infection (with the exception of PBG98, which induced a small subset of ISGs: *ENSGALG00000026970*, OAS*A and *β*-2M). This correlates with the characteristic ability of the poxviruses to modulate host innate responses [36]. Functional analysis of prototypical innate immunity and inflammation pathways of cESC confirmed suppressed responses (Supplementary files 1 and 4). For example, FP9 suppresses the expression of *IKBKE* (IKK-ε), which phosphorylates inhibitors of NFκB, by 8-fold, consequently suppressing expression of the proinflammatory cytokines *IL6* and *IL8* (by 11- and 14-fold, respectively). Furthermore, FP9 reduced the expression of *TRAF3* and *TRAF7* (both by approximately 2-fold). The proteins encoded by these transcripts are members of the TNF receptor associated factor (TRAF) protein family, which associate with and mediate the signal transduction cascades that lead to activation of transcription factors such as NFκB and the interferon-regulatory factors that results in induction of immune and inflammatory responses [40]. MVA suppressed the expression of *NFκB*, *IL8*, the RNase L inhibitor (*RLI*), the translation initiation factors *EIF2S1* and *EIF2B2*, and apoptotic adaptor *FADD* transcript. Taken together, these results suggest that cESC IFN and inflammatory responses are efficiently blocked by the viruses. One difficulty in interpreting these results is that the cell population is heterogeneous. Without recourse to single-cell sequencing, it is therefore difficult to determine whether subpopulations exist that are responsible for conferring specific host responses and/or permissivity/restriction to viral replication.

### 3.6. Correlation Analysis between RNA-seq and qRT-PCR Analyses

RNA-seq data were validated by quantifying the mRNA abundance of selected transcripts (*GAPDH*, *SOCS1*, *Mx1*, *IFIT5*, *PYURF*, *NANOG*, *HDAC2*, OAS*A, *TRAF3*, *ISG12(2)*) using qRT-PCR (Supplementary file 5). Pearson’s correlation test was performed to test for pairwise correlations among the two methods on all genes. The correlation coefficients (*r*) for all comparisons were over 0.99, indicating the high reproducibility of results with both methods.

The availability of cESC as a cell-line that supports poxvirus replication also enables further in vitro exploration of the virus replication, for instance to identify host-range genes. The ability of cESC to differentiate to different cell types can also be explored in order to investigate lineage-specific viral tropism. Once the host genes needed for virus replication are identified, clustered regularly interspaced short palindromic repeat (CRISPR)-*Cas9* reagents could be used to knock them out, to improve viral antigen production and/or increase viral replication with the long-term goal of creating more virus-permissive cell lines. These ‘enhanced’ cell lines will have potential applications in diagnosis and vaccine production. Altering the host innate immune response via modulating key innate immunity regulators (e.g., intracellular SOCS1) might also offer another simple and flexible solution to enhance viral propagation in culture, especially using IFN-sensitive viruses [30]. We are intrigued that cESC are restrictive to the “mild”, CEF-adapted IBDV vaccine strain PBG98. Future work should clarify whether this restriction extends to other IBDV strains, such as the “intermediate” attenuated Vero cell-adapted D78 and the “very virulent” strain UK661 (which can only be propagated in vivo or in ex vivo cells derived from the bursa of Fabricius [41]).

In conclusion, we have examined cESC in the context of stimulation with recombinant chIFN-α or infection with various vaccine vector viruses, showing that cESC maintain prolonged productive infection of poxvirus vaccine vectors. Our work demonstrates the utility of cESC as a high-yielding cell line for the propagation of vaccine or other viruses and as a biologically relevant platform to study the molecular pathobiology of avian viruses.

## Figures and Tables

**Table 1:** Matrix showing differentially expressed transcripts identified from RNA-sequencing analysis of cESC following treatment (chIFN-α-stimulation or virus infected). Comparisons were normalised against respective mock controls. Numbers of significant genes are indicated for fold change in expression ≥±1.5 and ≥±3 and for false discovery rate (FDR) ≤0.05 (Unpaired T-test with Asymptotic *p*-value computation and Benjamini–Hochberg Multiple Testing correction).

Comparisons vs. Respective	# Upregulated Genes		# Downregulated Genes	
Mock (FDR < 0.05)	≥1.5	≥3	≥−1.5	≥−3
chIFN-α 6 h	534	278	412	238
FP9 16 h	98	32	116	39
CNPV 16 h	164	86	1403	693
PBG98 16 h	13	5	26	5

## Supplementary Materials

The following are available online at https://www.mdpi.com/2073-4425/10/3/237/s1. Supplementary file 1: Spreadsheet workbook (.XLS) with worksheets showing full list of differentially regulated genes determined by six comparisons of nine different conditions analysed by RNA-sequencing. (a) chIFN-α stimulated versus mock-stimulated cESC, (b) FP9-infected versus mock-infected cESC, (c) MVA-infected versus mock-infected cESC, (d) CNPV-infected versus mock-infected cESC and (e) CNPV-infected versus mock-infected cESC and (f) chIFN-α stimulated versus mock-stimulated CEFs [from a previous analysis [25]]. Supplementary file 2: Gene set enrichment analysis (using Metacore GeneGO) for the RNA-sequencing data. Spreadsheet workbook (.XLS) with worksheets showing enrichment analysis by (a) pathway maps, (b) process networks, (c) GO processes, and (d) protein function. Supplementary file 3: List of qRT-PCR primers used in the study. Supplementary file 4: Innate immune response to RNA viral infection. Illustration generated with the MetaCore GeneGO pathway analysis tool (Clarivate Analytics) and enriched with the differentially-regulated transcripts of the study associated with this canonical gene network. A description of the map symbols is in www.portal.genego.com. The red and blue bars (thermometers) illustrate expression levels of up- and down-regulated transcripts, respectively. Numbers in black circles (bottom of bars) indicate the comparison from which the transcript was identified as differentially-regulated. Supplementary file 5: Comparison of median expression levels for 10 selected transcripts determined by RNA-sequencing and qRT-PCR analysis. Plots show the log_10_ expression levels for 10 selected genes. Pearson correlation coefficient (*r*) is shown in the lower right corner.
