# Supplementary material for: Chicken Embryonic-Stem Cells Are Permissive to Poxvirus Recombinant Vaccine Vectors
Source: Genes (Basel). 2019 Mar 20;10(3):237. doi: 10.3390/genes10030237 (PMC6471371; doi:10.3390/genes10030237)
Supplement: Supplementary file 1 [file genes-10-00237-s001.zip › Suppl. material3.docx]

| **Name** | **Sequence (5’-3’)** | **Primer role** |
| --- | --- | --- |
| **cRS17** | CCCGCTGGATGCGCTTCATC | forward |
|  | ACACCCGTCTGGGCAACGAC | reverse |
| **cGAPDH** | GGCACTGTCAAGGCTGAGAA | forward |
|  | TGCATCTGCCCATTTGATGT | reverse |
| **cMx1** | CACACCCAACTGTCAGCGAT | forward |
|  | ATGTCCGAAACTCTCTGCGG | reverse |
| **cISG12(2)** | TGACCAGAACGTCCACAAAGCCG | forward |
|  | ACCTGCTCCTGGACCGATGCTT | reverse |
| **cOAS*A** | AAGAACTGGGACTTGGTGGC | forward |
|  | CCTTCAGCTCCCAGACTGTG | reverse |
| **cSOCS1** | AAGACCGCATCCGCTGGCCTA | forward |
|  | AGGCTGAAACGCCCCGTCTGAAA | reverse |
| **cIFIT5** | TGCTTCACCAGCTAGGACTCTGC | forward |
|  | TGGCTTTTGCTCTGTCACCACTTTG | reverse |
| **cHDAC2** | CGGCGAAATGTTGCAGATCAC | forward |
|  | TTTGCTCCTTTGGTGTCCGT | reverse |
| **cTRAF3** | TCCTTGGCCCTAGTTTGTAATG | forward |
|  | TCTCAATGTTCTGTAAGGGTTCTC | reverse |
| **cOct4** | TGCAATGCAGAGCAAGTGCTGG | forward |
|  | ACTGGGCTTCACACATTTGCGG | reverse |
| **cSox3** | GTCGGGGTGGGCCAGAGGAT | forward |
|  | GCTGTTCATGCCCGGGTGCT | reverse |
| **cNanog** | TGCACACCAGGCTTACAGCAGTG | forward |
|  | TGCTGGGTGTTGCAGCTTGTTC | reverse |
| **cEsrrb** | ATCCCAGGGTTCTCCAATCT | forward |
|  | CAGTTGTAGGATGGCCAGGT | reverse |
| **cTrim-71** | CATCGTGGCTGACCGCAGCA | forward |
|  | CGACGATCCGGCGTGAGACG | reverse |
| **cSall4** | GTCCACTGCGGACCCCAACG | reverse |
|  | GGTGGAGAAGGCACGGCCAC | forward |
| **cCldn3** | GGGTGGTTTCGGTCAGCGGG | reverse |
|  | GATGCTGCACAGCCAGCCCA | forward |
| **Gata4** | TTCGACAGCCCCATGCTGCAC | reverse |
|  | AAATTCGATGTTGGCATGCCGGG | forward |
| **cSox2** | ACTCGGCCGCGAACAACCAG | reverse |
|  | GCCCCGAGCCGTTTGCTGAT | reverse |
| **IBDV VP4** | GGCTGGTCCCGGAGCATTCG | forward |
|  | GGTCCACGTTGGCTGCTGCT | reverse |
| **FPV168** | ACCTCAAACAACCTCATC | forward |
|  | GTTAATACTTGTGACTGCTG | reverse |
| **MVA A12L** | ATGCGATGGATGGTCAGATT | forward |
|  | ATGGTTTCTTTTTCCCAGCA | reverse |
| **CNPV IL10** | CAAGTACAATACATAACATCC | forward |
|  | GCATTCATGATTAATATATTGTCTTTAC | reverse |
