# Supplementary figures and images for: Chicken Embryonic-Stem Cells Are Permissive to Poxvirus Recombinant Vaccine Vectors
Source: Genes (Basel). 2019 Mar 20;10(3):237. doi: 10.3390/genes10030237 (PMC6471371; doi:10.3390/genes10030237)

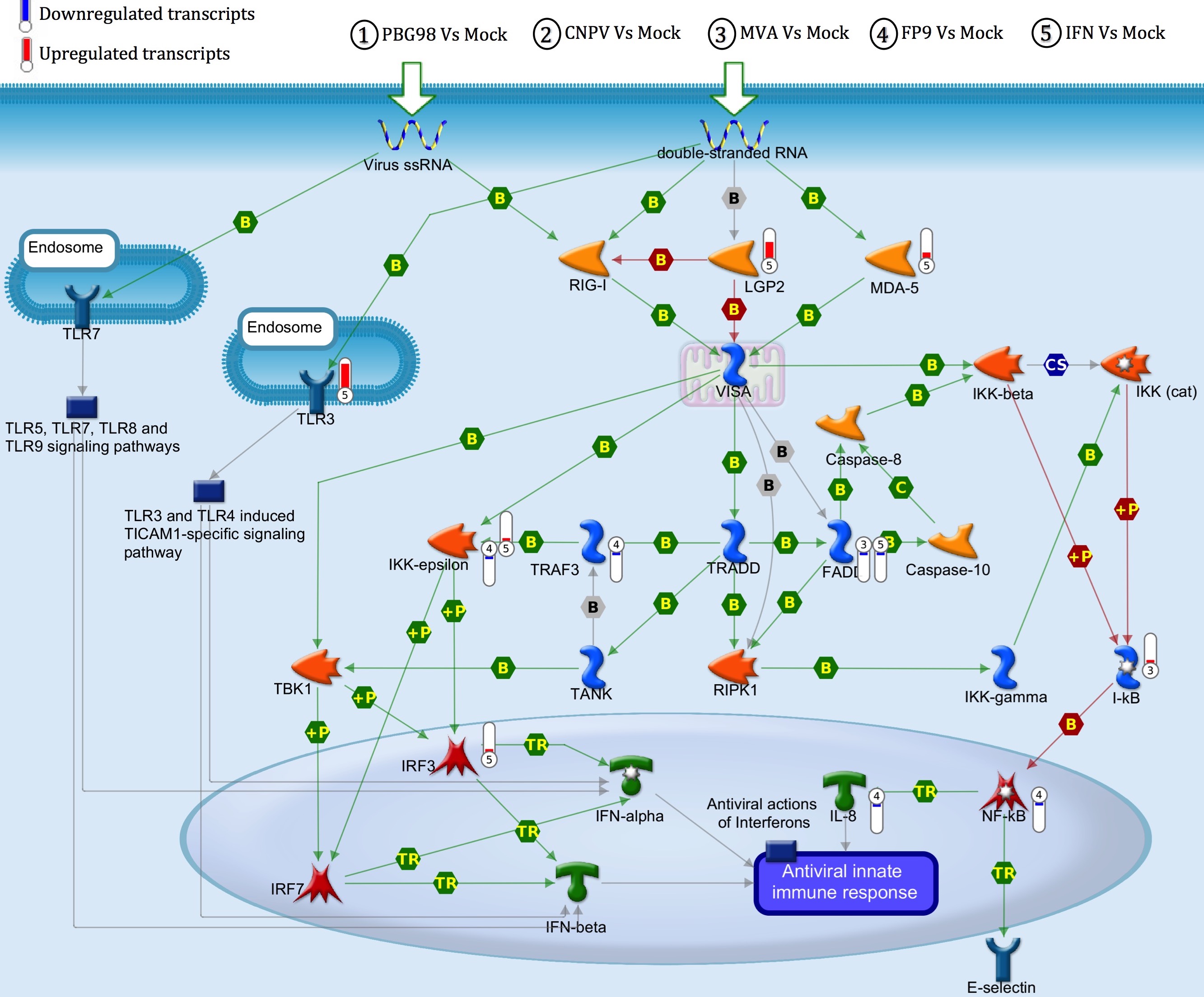

Supplement: Supplementary file 1 [file genes-10-00237-s001.zip › Suppl material4.jpg]

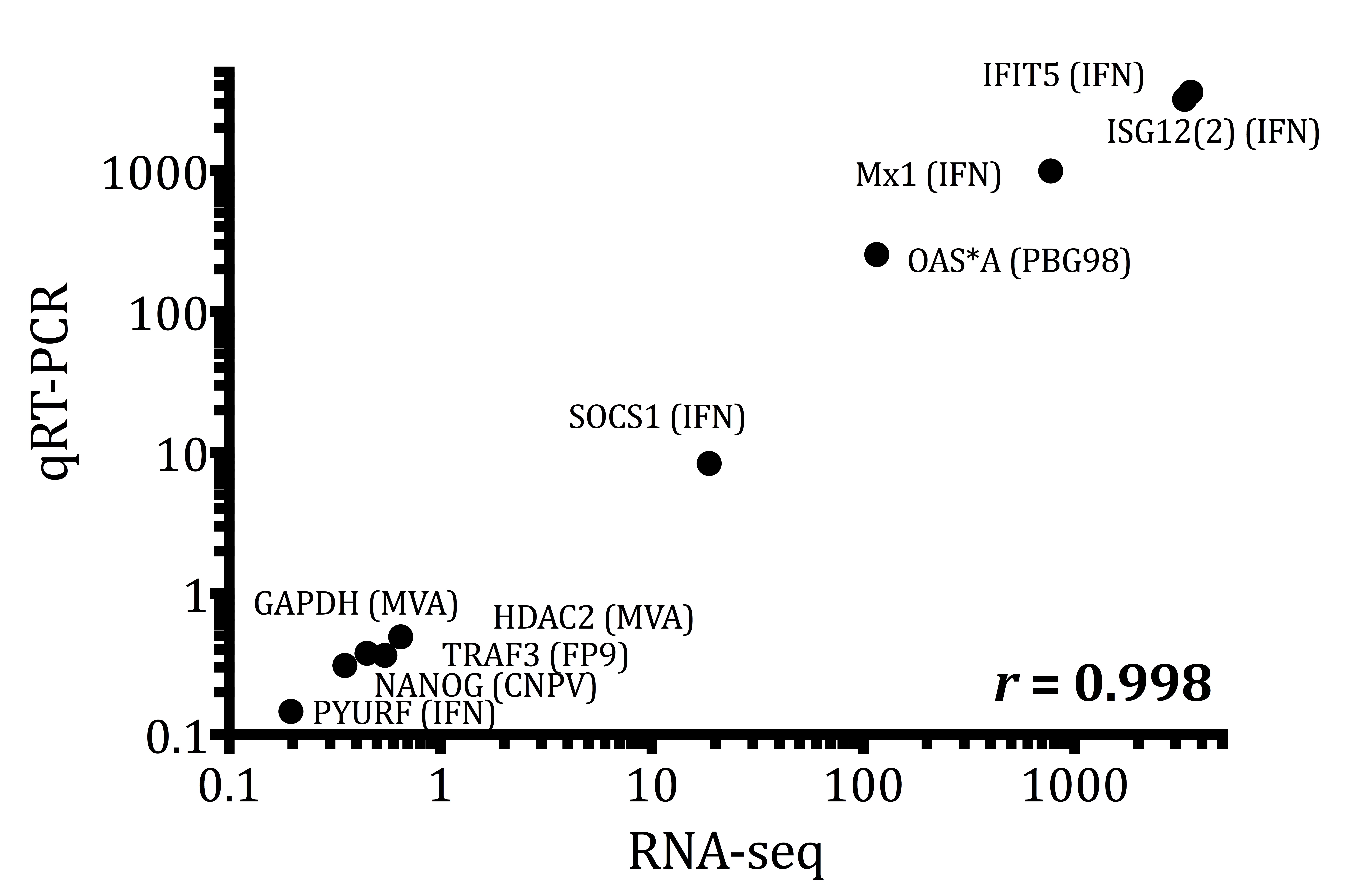

Supplement: Supplementary file 1 [file genes-10-00237-s001.zip › Suppl.material5.jpg]
